# Promoter Hypermethylation of Tumour Suppressor Genes as Potential Biomarkers in Colorectal Cancer

**DOI:** 10.3390/ijms16022472

**Published:** 2015-01-22

**Authors:** Jennifer Mun-Kar Ng, Jun Yu

**Affiliations:** Institute of Digestive Disease and Department of Medicine and Therapeutics, State Key Laboratory of Digestive Disease, Li Ka Shing Institute of Health Sciences, CUHK Shenzhen Research Institute, The Chinese University of Hong Kong, Hong Kong, China

**Keywords:** colorectal cancer, tumour suppressor gene, promoter methylation, CpG islands, biomarkers

## Abstract

Colorectal cancer (CRC) is a common malignancy and the fourth leading cause of cancer deaths worldwide. It results from the accumulation of multiple genetic and epigenetic changes leading to the transformation of colon epithelial cells into invasive adenocarcinomas. In CRC, epigenetic changes, in particular promoter CpG island methylation, occur more frequently than genetic mutations. Hypermethylation contributes to carcinogenesis by inducing transcriptional silencing or downregulation of tumour suppressor genes and currently, over 600 candidate hypermethylated genes have been identified. Over the past decade, a deeper understanding of epigenetics coupled with technological advances have hinted at the potential of translating benchtop research into biomarkers for clinical use. DNA methylation represents one of the largest bodies of literature in epigenetics, and hence has the highest potential for minimally invasive biomarker development. Most progress has been made in the development of diagnostic markers and there are currently two, one stool-based and one blood-based, biomarkers that are commercially available for diagnostics. Prognostic and predictive methylation markers are still at their infantile stages.

## 1. Introduction

Colorectal cancer (CRC) is the third most common cause of cancer in men and the second in women worldwide, with an estimated 1.36 million total number of new cases in 2012 [1]. It remains as the fourth most common cause of cancer death in the world (2008 and 2012), accounting for 8.5% of all cancer deaths and approximating to 694,000 deaths annually [1,2]. These high incidences of morbidity and mortality in CRC, unfortunately, have witnessed no improvement from 2008 to 2012 [1,2]. It can be, at least, partly explained by the rapid progression of disease and late clinical manifestations, which thus renders curative treatment impossible. Since the majority of colorectal cancers are sporadic but by and large preventable upon early diagnosis, the significance of screening and early detection cannot be clearer [3,4].

Building on the 1990 Vogelstein model, it is now recognised that colorectal cancer is driven by the accumulation of genetic abnormalities, through mutations and genomic instabilities, as well as epigenetic alterations [5]. In carcinogenesis, these changes often lead to gain of function in oncogenes or loss of function in tumour suppressor genes [6]. As they normally play a regulatory role in various conserved signalling pathways, deregulation of these genes allows normal cells to evolve progressively to a neoplastic state through the acquisition of a succession of the six hallmark capabilities [7]. Increasing evidence suggests that in the average CRC genome, many more genes are affected by aberrant epigenetic alterations than genetic mutations [8]. Additionally, only approximately 15 genetic mutations contribute to CRC pathogenesis with the majority reported to be passenger mutations that provide no selective advantage [9]. Epigenetic alterations are heritable changes in gene expression that are not as a result of changes in the DNA sequence. Those believed to have a role in cancer development include DNA methylation of CpG islands, histone modifications, microRNAs and noncoding RNAs, and nucleosome positioning [10]. In particular, hypermethylation of gene promoters enriched with CpG dinucleotides can induce transcriptional silencing of tumour suppressor genes [11]. Recent advances in epigenetics have not only provided insights and a deeper understanding of their role in CRC carcinogenesis, but have also led to the development of potential novel biomarkers.

It follows that the development of diagnostic biomarkers could help clinicians in earlier disease identification and in the administration of treatment and professional support [3]. There is a significant amount of interest in developing biomarkers for prognosis and surveillance as well as those that are predictive of cancer treatments [9]. Furthermore, the identification of cell-free nucleic acid in various bodily fluids and stool increases the possibility of detecting methylated biomarkers noninvasively [3]. Taken together, these biomarkers present a powerful approach to improve on the current diagnostic techniques and to optimise therapeutic decision-making, ultimately to achieve earlier diagnosis and lower the number of CRC morbidity and mortality.

In this review, we would like to provide an overview of the relationship between DNA methylation and colorectal cancer. In this context, we focus on hypermethylation, hypomethylation and a subset of CRCs called CIMPs. We also discuss the translation of epigenetic research into the development of potential DNA methylation-based biomarkers as diagnostic, prognostic and predictive tools in the clinic, as well as explore the myriad of technologies that are frequently used to detect these methylation biomarkers.

## 2. DNA Methylation and Colorectal Cancer

### 2.1. DNA Hypermethylation

DNA methylation is an enzymatic process involving the addition of a methyl group to the 5'-position of the pyrimidine ring of cytosines to produce 5-methylcytosine. This covalent modification is catalysed by DNA methyltransferases (DNMTs) in CpG dinucleotide sequences concentrated in short CpG-rich DNA stretches known as CpG islands and regions of large repetitive sequences including centromeric repeats and rDNA [12]. CpG islands overlap the promoter region of 60%–70% of genes and lack methylation in normal mucosa, independent of the transcriptional status of the gene. Hence, if this safeguard is lost, the promoters may become aberrantly hypermethylated and as a result induce transcriptional repression. This can be achieved through multiple mechanisms, including directly inhibiting cis-binding elements, such as the transcription factors AP-2, CREB, E2F, CBF and NF-κB, from accessing their target-binding site [5,13,14,15,16,17]. Alternatively, methylation can induce a compact chromatin structure by providing additional binding sites for methyl-binding proteins, which leads to repression of gene expression through interactions with histone deacetylases [18].

In CRC, promoter hypermethylation-induced inactivation of tumour suppressor genes has been observed at each histological step of the polyp to adenocarcinoma sequence [5]. Evidence from the last decade has indicated that epigenetic alterations do in fact have a pathological role in CRC and is not merely a consequence or by-product of malignancy [19,20]. Extrapolation of current data also estimates that aberrant promoter methylation is initiated at ~1% of all CpG islands and as much as 10% become methylated during the multistep process of tumorigenesis [21,22]. Furthermore, it is now understood that epigenetic alterations can also act as one of the “two hits” necessary for biallelic silencing of tumour suppressor genes without loss of heterozygosity as initially suggested by Knudson’s two-hit model [23,24]. Hence, hypermethylation events at CpG islands functions equivalently to coding-region mutations or deletion which can affect virtually all signalling pathways, including those of TP53, TGFβ/SMAD, WNT, NOTCH and receptor tyrosine kinases as well as those involved in cell cycle regulation, transcription regulation, DNA stability, apoptosis, cell-to-cell adhesion, angiogenesis and invasion and metastasis [9,25]. Naturally, promoter hypermethylation permits normal cells to acquire a succession of hallmark capabilities that enable them to become tumorigenic and ultimately malignant. Many genes have been reported to be methylated and silenced in CRC, some commonly methylated ones include *APC*, *MLH1*, *MGMT*, *SFRP1*, *SFRP2*, *CDKN2A*, *TIMP3*, *VIM*, *SEPT*, *CDH1* and *HLTF*.

Many risk factors are demonstrated to interact with epigenetics in tumours. Most of the studied risk factors are from a dietary perspective and they include high levels of processed meat, low folate and fibre intake and high levels of alcohol. Low folate levels in blood are associated with increased risk of LINE-1 hypomethylation. (discussed below) [26,27]. Aside from risk factors causing epigenetic alterations, they can also vary in different subtypes of CRCs. In Lynch syndrome for instance, aberrant methylation of intron 1 is frequently observed at the adenoma stage. Sporadic tumours present a more complicated case whereby consideration must be paid to site-dependence and mutual exclusivity of mutations in the *KRAS* and *BRAF* genes and the crosstalk between epigenetics and genetics [28,29].

#### 2.1.1. CpG Island Methylator Phenotype (CIMP)

In addition, there is a distinct subset of CRC, known as the CpG island methylator phenotype (CIMP), that is characterised by high frequency of promoter hypermethylation [5,30]. It exhibits distinctive molecular and clinicopathological features suggesting that CIMP represents a distinct carcinogenic pathway [31]. Quantitative DNA methylation analysis demonstrated that approximately 20% of CRCs are CIMP tumours, and there are significant associations with old age, female sex, proximal colon location, poor differentiation, *MSI* and *KRAS* and *BRAF* mutations [32,33]. Smoking is associated with increased risk of CIMP-high tumours [28]. The criteria to which they can be differentiated have been met with considerable controversy as the underlying cause of CIMP is still not well-understood. The most commonly used CIMP markers are *MLH1*, *p16*, *MINT1*, *MINT2* and *MINT31*. An extension has been made to include *CACNA1G*, *CRABP1*, *IGF2*, *NEUROG1*, *RUNX3*, *SOCS1*, *HIC1* and *IGFBP3* with no consensus on how many markers or which ones are required for positive CIMP identification [31]. Therefore it is not surprising that clinical results vary and may even be contradictory in many instances, depending on which criteria was used to define the CIMP subgroups [9,31]. CIMP can typically be divided into two subclasses: CIMP-high and CIMP-low. Genome-scale DNA methylation profiling of 125 CRC cases revealed that CIMP-high tumours have high *BRAF* mutations (61%), whereas CIMP-low tumours rarely have mutant *BRAF*, but is associated with high *KRAS* mutations (45%). This classification is similar to the findings by Shen *et al.* despite the different CIMP1 and CIMP2 naming. In addition to CIMP1 associated with high *BRAF* mutations, they were also microsatellite unstable [34,35,36,37,38].

CIMP tumours in CRC are hypothesised to develop from hyperplastic polyps (HPs) via the serrated pathway [39]. Serrated lesions include goblet cell HPs, microvesicular HPs, sessile serrated adenomas (SSAs) and traditional serrated adenomas (TSAs). SSAs have the characteristics of high CIMP, *BRAF* mutation and proximal colon location, and are precursors for CIMP-high CRCs [31]. Detection followed by removal of SSAs during colonoscopies would reduce CRC mortality, but the challenge lies in discriminating SSAs from HPs as neither show surface microstructure (pit pattern) specific to malignant lesions [31]. A 2013 population study reported the increased likelihood of CIMP tumours in CRCs diagnosed within five years after colonoscopy than those after that period or without endoscopy. This further supports the CIMP tumours observed in interval cancers, defined as CRCs that develop within five years of complete colonoscopy, which was found to be more prevalent (57%) in interval than in non-interval cancers (33%). Together, they reflect the difficulty of SSA detection during colonoscopy and supports the hypothesis that tumorigenesis that occurs in interval cancers (due to CIMP) may follow a different molecular pathway to non-interval cancers [40,41].

#### 2.1.2. DNA Methylation and miRNAs

Recently, it has been suggested that there may be complex interplay amongst the various epigenetic alterations in CRC, especially after silencing of tumour suppressive microRNAs (miRNAs) by promoter CpG methylation was reported [42]. miRNAs are small noncoding RNAs that have a role in post-transcriptional regulation of gene expression [43]. They bind complementary sequences on mRNA molecules to induce mRNA silencing often by proteosomal degradation, translational repression or deadenylation [44]. Accordingly, miRNAs may function as oncogenes or tumour suppressors depending on the genes that are inactivated. mir-17-92 for instance, is an oncogene as its overexpression promotes cancer development through negative regulation of tumour suppressor genes [45]. At the transcriptional level then, if mir-17-92 is methylation sensitive, then methylation may silence these oncogenic miRNAs to re-achieve physiological regulation of the tumour suppressor gene. Similarly, the converse is also true where DNA methylation-induced downregulation of tumour suppressive miRNAs contributes to CRC [46]. One of the mechanisms behind suppressed miRNA expression has been linked to overexpression of DNMTs which can facilitate increased DNA methylation [47,48]. Therefore, DNA methylation can directly induce miRNA transcriptional repression, which has the potential to indirectly cause further DNA methylation.

Thus far, the discussion has alluded to the association of hypermethylation with cancer, nevertheless, it is important to recognise that DNA methylation of CpG dinucleotides outside the promoter regions in mammalian cells is a normal mechanism. It provides a stable gene silencing mechanism that plays an important role in regulating gene expression and chromatin architecture [10]. Mammalian development, X-chromosome inactivation in females and genomic imprinting are heavily dependent on CpG island methylation [12]. Loss of imprinting (LOI) can result in the overexpression of either a paternally or maternally imprinted gene, such as *IGF2* or *H19*, which may lead to abnormal growth and increase susceptibility to cancer [49,50].

### 2.2. DNA Hypomethylation

Aside from the above mentioned promoter CpG island hypermethylation, global DNA hypomethylation in non-promoter regions is also often observed in CRC tumorigenesis. It manifests as an early event in the pathogenesis of cancer in an age-dependent manner. Global DNA hypomethylation occurs mostly at repetitive sequences including LINE-1 repeats, retrotransposons, introns and gene deserts [5,10]. DNA hypomethylation plays an important role in genomic instability by disrupting chromosomal structure and integrity. This is exemplified by patients with immunodeficiency centromeric instability and facial abnormalities (ICF) syndrome believed to be induced by germline mutation of the DNMT3B enzyme (*de novo* methyltransferase) [51]. Moreover, hypomethylation-induced loss of imprinting (LOI) which results in the overexpression of imprinted genes such as *IGF2* has strong implications in the carcinogenesis of CRC [52]. Recently, Hur K *et al.* reported that hypomethylation of LINE-1, which encodes an additional internal antisense promoter, inadvertently activated evolutionarily methylation-silenced proto-oncogenes such as *MET*, *RAB3IP* and *CHRM3* [53].

## 3. Technologies for the Identification and Routine Analysis of DNA Methylation Markers

Identification of genome-wide DNA methylated markers can be broadly summarised into two main steps. The first, known as pre-treatment, often captures the methylated cytosine residues which permits differentiation from the unmethylated; the latter permits visualisation and subsequent analysis of these methylated CpG sites. One of the more common approaches to pre-treatment is sodium bisulphite conversion of genomic DNA. This method relies on the hydrolytic deamination of denatured genomic DNA, specifically leading to the conversion of cytosine to uracil, whilst leaving 5-methylcytosine nonreactive under the chosen conditions. During subsequent PCR amplification of the bisulphite-treated DNA, uracil is replaced by thymine and 5-methlycytosine amplifies as cytosine [54]. The two approaches of the subsequent DNA methylation analysis step, either methylation-specific PCR (MSP) or non-MSP, dictates how the PCR primer should be designed. MSP uses primers that are designed to anneal specifically with either the methylated or unmethylated version of the bisulphite-converted sequence and it excels at the sensitive detection of methylation patterns. The alternative approach of the non-MSP method, which is more suitable for quantitative or detailed analysis, combines bisulphite conversion with sequencing (BGS), combined bisulphite restriction analysis (COBRA) and methylation-sensitive single-nucleotide primer extension (Ms-SNuPE) or several other techniques. The primers for non-MSP amplification do not cover any potential DNA methylation sites, and since bisulphite-converted DNA is non-self-complementary, the forward and reverse primers will differ [55]. Several other methods apart from the use of sodium bisulphite have been developed, this includes combining methylation-sensitive (HpaII) and insensitive (MspI) restriction enzyme digestion with the HELP assay and the use of MeDIP following the capture of methylated genomic DNA fragments using a 5-methylcytosine specific antibody. Ultimately, choosing the most suitable method depends on the application, required data resolution, genome size, cost and throughput in the number of samples [56].

Aside from the common combinations described above, the various methods used to analyse genome-wide DNA methylation patterns can, in effect, be combined with a variety of microarray and sequencing-based read outs. Whole-genome bisulphite sequencing (WGBS) is the current gold standard for the genome-wide identification of differentially methylated regions at single nucleotide resolution [21]. However, due to its high sequencing cost and requirement of substantial DNA quantities [57], other methods such as reduced representation bisulphite sequencing (RBBS), methylated DNA immunoprecipitation (MeDIP) and tagmentation-based whole-genome bisulphite sequencing (T-WGBS) [21,57]. T-WGBS requires less than 20 ng of DNA from biological samples and yet permits comprehensive methylome analysis [57]. With the advent of next-generation sequencing, numerous other methods have been developed with improved sensitivities and potential increase in cost-effectiveness [58]. Promising results have been reported in cancer detection using techniques such as bisulphite-seq, which couples bisulphite treated genomic DNA with next-generation sequencing [59], and methyl-BEAMing [60]. Methyl-BEAMing yielded a sensitivity that was four times higher than that obtained by assaying serum-carcinoembryonic antigen (CEA). It was successful in the detection of advanced adenomas in both plasma and faecal samples [60]. Alternatively, epigenotyping technologies such as the Infinium Human Methylation 27 K or 450 K BeadChip (Illumine Inc., San Diego, CA, USA) is a high-throughput platform that allows the methylation state of 27,000 to 450,000 CpGs to be assayed and analysed [61,62,63]. When compared to sequencing-based technologies, it has the advantage of providing straightforward bioinformatics data analyses and does not require correction for CpG density. However, microarrays do require adapted normalisation protocols for the two different array chemistries [21,62,64].

The above techniques cover how DNA methylation levels may be analysed, but it is necessary to first build a methylation profile to identify those that play a role in the pathogenesis of CRC. Methylation-sensitive arbitrarily primed-polymerase chain reaction (MS-AP-PCR) among other genome-wide screening methods can be used to analyse methylation differences between two groups of clinical DNA samples, e.g., between CRC tissue and equivalent histologically normal tissue from the same patient [51,55]. MS-AP-PCR works by comparing the positions of the gel-spot patterns of the sample tissues after PCR on polyacrylamide gel in order to rapidly identify the CpG islands that are differentially methylated [51].

The recent advances in methylation biomarker development are complemented by the DNA-methylation-based technologies that can potentially be used in routine diagnostics. Ideally, they must be cost-effective, sensitive and specific, and have a quick results turnover. Furthermore, to avoid the risk of cross-contamination and their resulting false-positives, closed tube assay formats are preferred. Most biomarkers must be detectable in specimens collected through minimally invasive procedures and hence, they tend to be DNA extracted from bodily fluids or tissue samples. Pyrosequencing, primer extension and real-time qPCR are suitable for quantitative analysis of DNA extracted from primary tissue and also for monitoring CRC recurrence in patients who have undergone resection. Detection of DNA methylation in body fluids is comparatively more challenging as only relatively low concentrations of target DNA are present. Technologies that are applicable require particularly high specificity and sensitivity, thus are mainly real-time PCR based approaches: MethyLight [65], HeavyMethyl [66] and methylation-sensitive melting analysis after real-time methylation specific PCR (SMART-MSP) [21,67]. In 2010, He *et al.* developed a multiplex MethyLight PCR assay which combines the specificity of real-time PCR, the high throughput of multiplex PCR and the high sensitivity of multigene detection into one assay. It takes into account all the parameters of clinical application and is a potential candidate for future biomarker screening programs [68].

## 4. Promoter Hypermethylation as a Biomarker

The widespread occurrence of epigenetic alterations in CRC has broad potential for important clinical applications. Their potentials as molecular markers are becoming increasingly attractive due to their inherent stability and pharmacological reversibility [69] on top of the need to build upon the inadequacy of the current diagnostic methods. Currently, colonoscopy and faecal occult blood testing (FOBT) represent the gold standard for CRC detection. Despite their success in reducing mortality in randomised controlled trials [70,71], half of all CRCs are only detected at the advanced stage. Coupled with some drawbacks of the invasiveness and associated risks of colonoscopy/sigmoidoscopy as well as the low specificity of FOBT, the need for more sensitive and specific non-invasive early diagnostic tools cannot be clearer [9]. Certainly, the possibility of minimally invasive procedures to identify methylation biomarkers in cell-free nucleic acid highlights further potential of these markers [3]. In addition to improving diagnosis, DNA methylation is also being explored for cancer risk evaluation, prognosis stratification and treatment response prediction [72].

### 4.1. Diagnostic Biomarkers

Since epigenetic alterations occur at a much higher frequency than genetic mutations, they may play a large role in the advancement of epigenetic markers as an early diagnostic tool, either used alone or to complement existing diagnostic methods [73]. Indeed, we must identify those that are methylated early in cancer e.g., in precancerous lesions [74] or early in the adenoma to adenocarcinoma sequence. This type of early detection screening may be pivotal, for sporadic cancers as well as in people with a family history of CRCs due to the epigenetic heritability, as the odds of survival are highest at this stage. Examples of genes methylated early and involved in the initiation of CRC include *SLC5A8*, *MINT1*, *MINT31*, *SFRP1*, *SFRP2*, *CDH13*, *CRBP1*, *RUNX3*, *p14ARF*, *HLTF*, *ITGA4*, *CDKN2A* (*p16*), *CDH1* and *ESR1* [5]. It is possible to detect promoter hypermethylation in DNA from various sources, including tissue biopsies, blood samples, stool, peritoneal fluid and urine. We will review biomarkers in blood and faecal samples as they’re more applicable to CRC.

#### 4.1.1. Early Detection in Blood

Benign and malignant lesions can increase the levels of circulating nucleic acids by up to 15-fold, where the concentration in patients with metastasised cancers can reach up to 500 ng/mL [21]. The substantial release of cell-free DNA is probably by apoptotic and necrotic cells and the tumour-related epigenetic alterations can be specifically quantified amongst the normal DNA [75]. Various genes including *hMLH1*, *CDKN2A* (*p16*), *HLTF*, *ALX4*, *TMEFF2*, *NGFR*, *NEUROG1*, *SFRP2* and *RUNX3* have emerged as potential blood-based methylation markers for CRC with sensitivities ranging from 34% to 90% and specificities spanning from 69% to 100% [76,77,78,79,80,81,82,83,84,85,86]. Hence, with the ease at which blood can be acquired, detection of blood-based biomarkers would provide a practical screening tool for CRC.

In fact, a blood-based assay that detects methylated septin 9 gene (*SEPT9*) has been commercialised by Epigenomics AG (Berlin, Germany) under the name Epi proColon^®^ and is currently being marketed in several countries. *SEPT9* codes for a GTP-binding protein implicated in cell division, cytoskeletal organisation and membrane remodelling [87]. The v2 region of the *SEPT9* promoter has been shown to be methylated in CRC tissue and not in the normal colonic mucosa. *SEPT9* has also been shown to be aberrantly methylated in lung and breast cancers, however, the rate of positive detection in plasma samples were not significantly different from controls [77]. It was identified amongst more than 600 candidate genes by MS-AP-PCR [88] and further validated by microarray and real-time PCR testing using plasma samples. *SEPT9* fulfilled the required criteria and produced the best results by achieving 95% specificity, 52% sensitivity and an AUC of 0.80 [76]. Subsequent test developments with real-time PCR further optimised *SEPT9* detection, achieving an overall sensitivity of 90% and specificity of 88%, thus warranting its use for blood-based CRC screening [77,78].

#### 4.1.2. Early Detection in Stool

Biomarker detection in stool represents another promising approach for CRC screening. From a patient’s point of view, the practicalities of stool testing far outweigh those of blood-based assays. They’re uniquely non-invasive, requires no formal visit to the clinic or time away from work. Hence, it is both safe and convenient for patients, and with a mailing-in service for specimens, patients can have widespread access to stool screening. However, much like the blood-based assays, technical challenges remain to recover analysable human DNA from stool. Human DNA represents a mere 0.01% of total stool DNA from abundant exfoliation of neoplastic epithelial cells lining the colon and rectum; the remaining 99.99% is nonhuman, either from microflora or diet [89]. Examples of a few promising stool-based biomarkers can be found in Table 1.

**Table 1:** DNA methylation biomarkers in blood and stool samples of colorectal cancer patients.

Biomarkers	Sensitivity (%)	Specificity (%)	References
***Blood Samples***			
*SEPT9* (commercially available biomarker)	72	90	[77]
	90	89	[78]
*hMLH1*	43	98	[82]
	33	100	[79]
*CDKN2A/p16*	71	100	[86]
	70	100	[80]
*HTLF*	34	98	[82]
*ALX4*	83	70	[83]
*TMEFF2/HPP1*	65	69	[76]
*NGFR*	51	84	[76]
*NEUROG1*	52 (stage I)	91	[84]
	64 (stage II)		
*SFRP2*	67	94	[85]
*RUNX3*	65	100	[86]
***Stool Samples***			
*VIM* (commercially available biomarker)	88	82	[90]
	72	89	[91]
	46	90	[92]
*SFRP2*	87	93	[93]
	77–90	77	[94]
*TFPI2*	76–89	79–93	[95]
*GATA4*	51–71	84–93	[96]
*NDRG4*	53–61	93–100	[97]
CDKN2A/MGMT/hMLH1 *	55	72	[98]
SFRP2/HPP1/MGMT *	96	96	[74]
ITGA4/SFRP2/CDKN2A *	70	97	[99]

*hMLH1*: Homo mutL homolog 1; *CDKN2A*: Cyclin-dependent kinase inhibitor 2A; *HLTF*: Helicase-like transcription factor; *ALX4*: ALX homeobox 4; *TMEFF2*: Transmembrane protein with EGF-like and two follistatin-like domains 2; *HPP1*: Hyperplastic polyposis 1; *NGFR*: Nerve growth factor receptor; *NEUROG1*: Neurogenin 1; *SFRP2*: Secreted frizzled-related protein 2; *RUNX3*: Runt-related transcription factor 3; *MGMT*: *O*-6-methylguanine-DNA methyltransferase; *TFPI2*: Tissue pathway inhibitor 2; *GATA4*: GATA binding protein 4; *NDRG4*: *N*-myc downstream regulator gene 4; *ITGA4*: integrin, alpha 4; * The values do not refer to comethylation.

A clinically validated stool-based assay under the name ColoGuard^®^ is currently commercially available in the USA. The test detects the hypermethylated vimentin gene (*VIM*) which encodes an intermediate filament protein with a primary role in stabilising cytoskeleton. It is not normally methylated in normal colonic epithelia, but is highly methylated in CRC cell lines, in 53%–83% of cancer tissues and in 46% of the stool of CRC patients. Early stool-based detection assays achieved 46% sensitivity and 90% specificity, comparing favourably with FOBT (14% sensitivity and 95% specificity) as well as with other stool-based biomarkers [92]. Another PCR-based detection assay reported 72.5% sensitivity and 86.9% specificity during methylated *VIM* detection, but showed that incorporating another marker for DNA integrity further improved sensitivity with approximately equalling sensitivities for patients in various TNM stages [90,100]. Since some methylated biomarkers, including *SFRP2* and *TFPI2*, have also produced relatively high sensitivity and specificity (Table 1). Hence, the introduction of a multigene faecal methylation panel has the potential to lead to even better outcomes [74].

Despite the availability of two commercially-developed clinical biomarkers for early detection, their use and popularity is still rather limited. This may be partly due to the insufficient sensitivity and specificity required for a diagnostic test, undermined by the limited knowledge of DNA methylation patterns, the molecular heterogeneity of cancer and the most predictive CpGs in a gene of interest [21]. Understandably, from a patient’s perspective, they would still prefer the current well-tested diagnostic tools over the uncertainties surrounding epigenetic biomarkers. In light of this, detection methods are constantly under development to enhance the specificity and optimise screening sensitivity of stool- and blood- based assays [25,100]. Indeed, from Table 1, there are several biomarkers, both in blood and stool samples, that have high enough sensitivities and specificities to challenge the two approved diagnostic biomarkers, but it must be noted that many of the higher values refer to the neoplastic state. Unsurprisingly, the sensitivities are lower in adenomas/polyps and inflammatory bowel diseases which would be the ideal stages for early detection to avoid the morbidity associated with surgery. Huang *et al.*, reported sensitivities ranging from 17% to 96% in the detection of *SFRP2/HPP1/MGMT* in various stages of CRC and precancerous lesions. (CRC: 96%, advanced adenoma: 80%, non-advanced adenoma: 64% and hyperplastic polyp: 38% [74]). Thus caution must be taken when interpreting these findings as evidence to support CRC screening for early detection of CRCs. Multiple improvements such as multigene detection has been suggested since comethylation is a common phenomenon in CRC neoplasia [91,101]. Moreover, as methylated *VIM* has also been detected in urine, this alternative method of detection presents another interesting area for further research [102].

Inevitably, widespread clinical use will demand the best methylation biomarker and certain findings lend support to the superiority of stool-based assays over blood-based ones. It is possible that abundant marker release by luminal exfoliation into stool occurs earlier than by vascular invasion into blood during the progression of CRC tumorigenesis [103]. It can also be speculated that the accuracy to which CRCs can be diagnosed through stool-based samples is higher, as human DNA extracted from stool is more likely to be from the colon neoplasia than possibilities of a metastasis or another primary tumour.

#### 4.1.3. Epigenetic Biomarkers *vs.* Genetic Biomarkers

In addition to epigenetic biomarkers, genetic biomarkers have also been extensively studied. Putative genetic biomarkers for CRC include *APC*, *KRAS*, *p53* and *BAT26* (Table 2), all of which can be detected in stool DNA [104,105]. *APC* and *KRAS* are frequently mutated oncogenes in Vogelstein’s model, while *p53* is often characterised by loss-of-function mutations [106]. *BAT26* acts as a microsatellite instability marker that is observed to carry deletions in CRC patients [104]. Much focus of recent molecular diagnostic tests has been on faecal samples. Similar to epigenetic stool testing, this test relies upon preservation of DNA in stool samples to allow detection of accumulating mutations in the biomarkers. The earliest of these studies focused on detecting single *KRAS* and *APC* mutations, but due to the heterogenetic nature of cancers, assays to target multiple genes were developed to improve sensitivity [105]. Dong *et al.*, Alquist *et al.* and several other groups have since successfully shown consistency in achieving high sensitivity using varying combinations of *APC*, *KRAS*, *p53* and *BAT26*. The reported sensitivities range from 52% to 91% and specificities from 93% to 97% [107,108,109,110,111]. Notably, the range of sensitivities for multigene targets in CRCs are more variable than those yielded from single modality methylation biomarker screening. Nevertheless, like the methylation markers, sensitivity and specificity can be expected to improve with technical refinements and varying combinations of current or new tumour markers. Further compounding the issue are cost effectiveness, lack of formal FDA approval and lack of strong evidence regarding the screening interval [112]. The cost has been reported to be higher than ColoGuard ($599) [113] and the only commercially available test, PreGen-Plus, has yet to be approved by the FDA [112]. It can be speculated that this perhaps may be due to the lack of evidence exploring the significance of a positive genetic biomarker detection with a subsequent negative colonoscopic result [114]. Furthermore, there are concerns that the test may yield false positive results due to the potential shedding of supracolonic aerodigestive cancer cells into stool [114]. Understandably, these limitations provide an opening for the exploration of DNA methylation biomarkers in attempt to overcome the obstacles encountered by genetic biomarkers.

**Table 2:** Genetic biomarkers in colorectal cancer patients.

Biomarkers	Specimen
*APC*	Stool DNA
*KRAS*	Stool DNA, tissue
*p53*	Stool DNA, colonic effluent, colonic DNA
*BAT26*	Stool DNA, tissue

*APC*: Adenomatous Polyposis Coli; *KRAS*: Kirsten rat sarcoma viral oncogene homolog; *p53*: Tumour protein p53; *BAT26*: microsatellite DNA. Reproduced with permission from [105].

### 4.2. Prognostic Biomarkers

Moreover, epigenetic profiles could be useful as biomarkers for the prognosis of cancer patients. They can provide information about the patient’s cancer outcome, malignant potential of the tumour and risk of tumour recurrence regardless of therapy. It can also provide a rough estimate of the decreased survival time [21]. Hence, it can be used alongside tumour-node-metastasis (TNM) staging, which is currently the most important clinical predictor of patient outcome [115], to guide re-evaluation of clinical management thereby avoiding loss of valuable time due to ineffective treatment. Physicians may, for instance, consider additional adjuvants or combination therapies such as chemotherapy or radiotherapy alongside resection surgery, for patients who have been tested positive for a prognostic biomarker [116]. Many studies have investigated the potential of candidate genes for prognostic use, and unsurprisingly, most of which are similar to the ones that have a high potential for diagnostic use. Promoter CpG methylation of *HLTF* and *HPP1/TPEF* were found to be correlated with tumour size, metastatic disease and tumour stage with multivariate analysis indicating strong association with poor outcome [117,118,119]. *CDKN2A* hypermethylation was observed in 25%–30% of tumours, but there was conflicting evidence of its effectiveness as a prognostic biomarker. It has been reported to be more prevalent in more advanced Duke’s stages [80] and multiple studies have reported *CDKN2A* methylation as an indicator of disease recurrence and reduced survival in patients [29,120]. On the contrary, three prospective studies have witnessed no association between hypermethylation and survival [121,122]. Given the strength and relative sample sizes (326 and 902 participants), it would be reasonable to suggest that *CDKN2A* is not suitable as a prognostic marker [28]. Other independent prognostic biomarkers that have been recently identified include *TBX5* and *DACT2*. Promoter CpG methylation of both genes were detected in primary tumours but not in normal controls and also demonstrated significant association with shortened survival [115,123]. Research in prognostic biomarkers is still in its infancy and with our current knowledge, there isn’t a candidate biomarker suitable for clinical development; many of them are dependent on the presence of other methylated markers or adjuvant treatment [124].

### 4.3. Predictive Biomarkers

Predictive epigenetic markers will allow prediction of a patient’s response to a given therapy and thus guide selection of optimal treatment regimens based on the individual methylation profile. This is particularly valuable for CRC given its heterogeneity and patients may be spared from the toxic side effects and cost of standard cytotoxic chemotherapy that has a potentially low chance of benefit [21,125]. Methylation profiling has been successful in identifying these predictive markers in response to targeted therapy in cancers of the breast, melanomas and gliomas [126,127,128]. In 2012, Ebert *et al.* reported that *TFAP2E* hypermethylation was associated with clinical non-responsiveness to chemotherapy in CRC. *TFAP2E* normally represses *DKK4* promoter activity through direct binding, hence, low or no *TFAP2E* expression leads to *DKK4* overexpression seen in CRC cell lines and cancer specimens. *DKK4* has previously been implicated in fluorouracil resistance, and it was confirmed with CRC cell lines that *DKK4* over-expression increased resistance and the introduction of *TFAP2E* successfully increased fluorouracil sensitivity. Further supporting this, patients with hypomethylation showed a six-fold higher probability of response [129]. For CRC, pharmacoepigenomics could prove powerful in the identification of epigenetic biomarkers that can be used for understanding response to chemotherapy and drug resistance before treatment. There is vast scope and potential for epigenetics in drug treatment and it would undoubtedly be a major benefit for individual patients as well as the healthcare system. However, this is a relatively new field of research and mechanisms that underlie inter-individual differences in drug responses remain to be elucidated [130].

## 5. What about Biomarkers for Global Hypomethylation and CIMP?

Although CIMP represent approximately 20% of CRC tumours, clinical advancement of CIMP biomarkers are yet to be developed. This hindrance comes as there is still much debate over the universally accepted criteria for CIMP phenotype classification and a lack of validation of the accuracy of the methylated gene panels for designating epigenotypes. Furthermore, there is considerable overlap between CIMP and sporadic microsatellite instability tumours which adds to the complexity of clinical trials and clinical decision making [5,131]. To date, at least 8 studies have been suggestive that CIMP may have a prognostic role in colorectal cancers [132,133,134,135]. Recently, Li *et al.* reported the association of high CIMP with poor prognosis and overall reduced survival in Northeast China patients. Patients with high CIMP presented a worse prognosis than those of no and low CIMP combined [136]. On the other hand, at least 15 other studies produced conflicting evidence and stated null association between CIMP and CRC prognosis with some even reporting better prognosis with CIMP [137,138,139,140]. Thus far, no conclusions can be drawn regarding the utilisation of CIMP as a prognostic biomarker. CIMP’s predictive potential has also been reported in retrospective studies, including its responsiveness to 5-fluorouracil [35,141]. Furthermore, CIMP tumours showed better response to the addition of the irinotecan (IFL) adjuvant to fluorouracil and leucovorin than non-CIMP tumours, which as a result also increased the patients’ survival times [142]. However, with conflicting evidence in the field, much like for CIMP prognostic markers, it is difficult to evaluate its use in predictive therapy.

Most research efforts have been focused on finding biomarkers for hypermethylated genes, justifiably, as its biological role of transcriptional silencing is more widely understood. However, the significance of global hypomethylation in CRC has been alluded to by the inverse correlation between CpG island methylation and global DNA hypomethylation which may represent other distinctive progression pathways in CRC [143,144]. LINE-1 hypomethylation is showing promise as a diagnostic biomarker for familial cancer risk assessment as well as a prognostic marker associated with poor survival outcome [145,146]. In 2013, Ogino *et al.* reported that individuals with a family history of CRC have a greater risk of developing tumours with lower LINE-1 methylation [145]. Moreover, evidence for prognostic potential comes from patient survival data of LINE-1 hypomethylation level as determined by pyrosequencing [115] and is further supported by the correlation between LINE-1 extreme hypomethylation and an earlier onset (<60 years) and poor prognosis of CRC [58,133]. Both MSI and CIMP CRCs depend on the methylation status of LINE-1. They have an inverse relationship which, at least in part, explains the poor survival as it suggests that increased LINE-1 hypomethylation serves as a significant prognostic parameter of adverse prognosis in MSI high CRCs [134]. Finally, low level LINE-1 methylation has the potential to be used as a biomarker of disease recurrence in resected stage II proximal CRC [133].

## 6. Conclusions and Perspectives

Promoter CpG island-mediated silencing of tumour suppressor genes plays an important role in the carcinogenesis of CRC. The identification of these methylated genes has been significant in the development of novel biomarkers as minimally invasive diagnostic, prognostic and predictive tools in attempt to increase efficiency and improve therapeutic outcome in the clinic. Promising data, as evident in the advancement of diagnostic markers, have been collected using newly-improved technologies that yield high sensitivity and specificity. Indeed methylation biomarkers exhibit huge potential for use in generalised population CRC screening, but as it stands, multiple hurdles remain to be addressed and overcome before translational epigenetics can be successfully implemented. Most of our knowledge lies in the downstream effects of epigenetics, and a deeper understanding of the causes and factors that undermine epigenetic alterations may be significant in explaining the differences between the healthy and diseased states. This should also extend to increasing the scope of knowledge on crosstalk between genetics and epigenetics along with the epigenetic variation in different subtypes of CRC which differ in a site-dependent and gene-specific manner [28]. The application of molecular pathological epidemiology (MPE) can potentially improve our understanding of epigenetics in this complex multifactorial disease. MPE is an emerging multidisciplinary field that examines each disease process from unique profiles of exposomes, epigenomes, transcriptomes, proteomes, metabolomes, microbiomes and interactomes in relation to the tissue microenvironment as well as the macroenvironment. Hence it allows methylation biomarkers, for instance, to be studied in relation to an exposure of interest; similarly, the effects of certain tumour epigenetic changes on prognosis may permit better understanding of the epigenetic changes and lead directly to biomarker discovery. The GWAS-MPE approach using epigenome-wide association studies is also another promising area [147,148].

Focusing on epidemiological limitations, most of the data in the current literature are limited by small sample numbers and poor controls [9]. As previously noted, caution must also be taken when interpreting evidence surrounding methylation biomarker studies as the data provided may not necessarily be sufficient for early diagnostic marker development. Therefore, multiple, prospective large-scale population studies and randomised clinical trials are urgently required to truly evaluate the clinical significance of these biomarkers by comparing the sensitivity, specificity and cost-effectiveness of biomarkers against current colonoscopy and FOBT. The increased sensitivity may allow differentiation of polyps with high malignant potential from the majority that are not precancerous, which can prevent unnecessary overtreatment as seen in prostate cancer screening [112].

Epigenetics is receiving greater attention than before, and the scope for this field in cancer is further exhibited by the development of miRNA biomarkers in colorectal cancer. To combat CRC heterogeneity and their use of multiple redundant signalling pathways, it is possible that using a combination of biomarkers may be a more effective tool in CRC. This can involve the coupling of methylation and miRNA biomarkers or methylation and DNA biomarkers. If the biomarkers used are all present in stool samples, a single stool specimen is sufficient to perform both evaluations in a non-invasive manner [112]. Imperiale *et al.* demonstrated that *BMP3* and *NDRG4* methylation biomarkers exhibited higher sensitivity at the expense of specificity with the *KRAS* genetic marker showing the opposite effect. Epigenetic and genetic markers therefore can complement each other to great effect in a stool-based approach [28,149]. This review has focused largely on diagnostic biomarkers; it echoes a clichéd yet meaningful quote “prevention is better than cure” whereby upon early diagnosis, progression into malignancy can be avoided altogether. Nevertheless, this ideal cannot always be achieved and the new emerging field of epigenetic therapy might prove useful for the cohort of patients with epigenetic alterations in CRC or with CIMPs [116]. Undoubtedly, many challenges and obstacles lay ahead in the epigenetic field, but the potential impact and contribution of these biomarkers to colorectal cancer, be it individual lives or the wider population, is monumental.
